# Contribution of an instructional module for lymph node evaluation: An experiment*

**DOI:** 10.1590/1518-8345.4166.3408

**Published:** 2021-04-12

**Authors:** Francine Lima Fulquini, Cristina Mara Zamarioli, Bárbara Gadioli, Luciana Kusumota, Fernanda Raphael Escobar Gimenes, Emília Campos de Carvalho

**Affiliations:** 1 Universidade de São Paulo, Escola de Enfermagem de Ribeirão Preto, PAHO/WHO Collaborating Centre for Nursing Research Development, Ribeirão Preto, SP, Brazil.; 2 Scholarship holder at the Coordenação de Aperfeiçoamento de Pessoal de Nível Superior (CAPES), Brazil.

**Keywords:** Teaching, Physical Examination, Lymphatic System, Teaching Materials, Simulation Training, Nursing, Ensino, Exame Físico, Sistema Linfático, Materiais de Ensino, Treinamento por Simulação, Enfermagem, Enseñanza, Examen Físico, Sistema Linfático, Materiales de Enseñanza, Entrenamiento Simulado, Enfermería

## Abstract

**Objective:**

to evaluate the contribution of an instructional module identifying the number and characteristics of lymph nodes by undergraduate nursing students.

**Method:**

an experimental, randomized, controlled and masked study using an instructional module for intervention. The 68 students who made up the control group or the experimental group performed the prototype lymph node palpation in the initial and final phases, following the free registration of the characteristics and number of these structures. Between the phases, the instructional module (palpation prototype and a registration guide instrument) was applied to the experimental group. Descriptive statistics and mixed linear regression were used for analysis.

**Results:**

the experimental group showed greater accuracy (p<0.05) in the evaluation of the size, consistency, mobility and coalescence of the lymph nodes in the final phase when compared to the control; it also showed more chances to correctly evaluate the consistency ( OR 45,26; 95% CI<7,74>‡<264.54> p<0.0001), mobility (OR 55.95; CI 95% 12.45 - 251.60; p<0.0001) and size (OR 25.64; CI 95% 3.92 - 160.2; p=0.0002) of the lymph nodes.

**Conclusion:**

the results reinforce the contribution of the instructional module to increase the knowledge of nursing students about the evaluation of lymph nodes.

## Abstract (pt)

**Objetivo:**

avaliar a contribuição de um módulo instrucional para a identificação do número e das características de linfonodos por estudantes de graduação em enfermagem.

**Método:**

estudo experimental, aleatorizado, controlado e mascarado utilizando para intervenção um módulo instrucional. Os 68 estudantes que compuseram o grupo controle ou o grupo experimento realizaram nas fases inicial e final a palpação dos linfonodos em protótipo, seguindo-se o registro livre das características e número dessas estruturas. Ao grupo experimento foi aplicado, entre as fases, o módulo instrucional (protótipo de palpação e um instrumento-guia de registro). Para análise empregou-se estatística descritiva e regressão linear mista.

**Resultados:**

o grupo experimento apresentou maior acerto (p<0,05) na avaliação do tamanho, consistência, mobilidade e coalescência dos linfonodos na fase final quando comparado ao controle; ainda, apresentou mais chances de avaliar corretamente a consistência (OR 45,26; IC 95% 7,74 - 264,54; p<0,0001), mobilidade (OR 55,95; IC 95% 12,45 - 251,60; p<0,0001) e tamanho (OR 25,64; IC 95% 3,92 - 160,62; p=0,0002) dos linfonodos.

**Conclusão:**

os resultados reforçam a contribuição do módulo instrucional para o aumento do conhecimento de estudantes de enfermagem sobre a avaliação de linfonodos.

## Abstract (es)

**Objetivo:**

evaluar la contribución de un módulo instruccional a la identificación del número y características de ganglios linfáticos por estudiantes de pregrado de enfermería.

**Método:**

estudio experimental, aleatorizado, controlado y enmascarado mediante intervención de un módulo instruccional. Los 68 estudiantes que integraron el grupo control o el grupo experimental realizaron en las fases inicial y final la palpación de los ganglios linfáticos en prototipo, tras el registro libre de las características y número de estas estructuras. El módulo instruccional (prototipo de palpación y un instrumento-guía de registro) se aplicó al grupo de Experimental. Para el análisis, se utilizó estadística descriptiva y regresión lineal mixta.

**Resultados:**

el grupo Experimental mostró mayor acierto (p<0,05) en la evaluación del tamaño, consistencia, movilidad y coalescencia de los ganglios linfáticos en la fase final en comparación con el control; aun así, presentó más probabilidad de evaluar correctamente la consistencia (OR 45,26; IC del 95% 7,74 - 264,54; p<0,0001), la movilidad (OR 55,95; IC del 95% 12,45 - 251, 60; p<0,0001) y tamaño (OR 25,64; IC 95% 3,92 - 160,62; p=0,0002) de los ganglios linfáticos.

**Conclusión:**

los resultados refuerzan el aporte del módulo instruccional para el incremento del conocimiento de los estudiantes de enfermería sobre la evaluación de ganglios linfáticos.

## Introduction

The practice of physical examination by nurses has been recognized as a relevant part of the care process^(^ 1 ^)^, given that it allows for the construction of clinical reasoning in the identification of health problems or conditions presented by the patient, as well as it assists in the choice of interventions to achieve the objectives being established for their recovery or health maintenance.

However, we have experienced the partial development of this procedure in the clinical practice, despite the fact that for decades the national literature has already highlighted the concern with the clinical assessment taught to future professional nurses and the need to prepare faculty to teach this theme, including culminating in actions aimed at raising such skills in the professors^(^ 2 ^)^. Equally deficient is the quality of the records regarding the physical examination performed by nurses^(^ 3 ^)^.

Considering that the skills and competences necessary to obtain data on the physical examination are dependent on the teaching strategies, we are interested in contributing to the updating of the contents taught, directly related to the students’ performance expectations, as the literature alerts^(^ 4 ^)^.

A more recent study also points out several obstacles to the physical examination, which can be related to “the precariousness of work, failures in the training process of the components of the nursing team, the organization of their work routines or even operational difficulties in service management”^(^ 5 ^)^.

Among the difficulties reported by nursing students for performing the physical examination, obstacles in the training process stand out, such as the short time of theoretical and practical classes in laboratories, and the low value attributed to this stage of data collection^(^ 6 ^)^.

In view of the complexity and importance of the physical examination for the practice of health disciplines, different strategies have been used for its teaching^(^ 7 ^-^ 8 ^)^; among those recommended for nursing courses, the early insertion of practice in the curriculum is recommended, including the adoption of simulation scenarios and the establishment of innovative teaching methodologies^(^ 9 ^)^.

Teaching new content and new practices should be challenging to allow students to advance their knowledge and skills^(^ 10 ^)^; for such, the active methodology is proven to be an effective teaching strategy^(^ 11 ^)^, favors the teaching-learning process^(^ 12 ^)^, reduces the patient’s exposure to risks^(^ 13 ^)^ and guides the development of skills, clinical reasoning and decision-making^(^ 14 ^)^.

Learning through experiences, built through stages that involve task accomplishment, exercise, reflection and application in other contexts^(^ 15 ^)^, is consistent with the purposes of this study, using simulators.

Given that new teaching strategies must be evaluated from the point of view of their effectiveness in order to be put into practice, this study had the purpose of contributing to the reflection on the teaching of physical examination, especially of the lymphatic system, in our view, one of the more complex and relevant. Complex because, in general, lymph nodes are found individualized, not palpable, they are small, free, painless structures, of fibroelastic consistency and that can be confused with other body structures^(^ 16 ^)^. Its relevance rests on the possible health changes that are related to the appearance of lymph node alterations. These are of varied origins and can be contemplated in mnemonic MIAMI (malignant diseases, infections, autoimmune diseases, miscellaneous or unusual conditions and iatrogenies) or CHICAGO (cancer, hypersensitivity, infection, collagenosis and other rheumatological diseases, atypical lymphoproliferative diseases, granulomatosis and other diseases)^(^ 16 ^-^ 19 ^)^.

It is therefore understood that the information obtained by the clinical examination as to the identification and characteristics of the lymph nodes, as well as the respective documentation, is essential for diagnostic and therapeutic clinical reasoning, which cause the teaching of this theme to be essential for safe and quality care.

This study aimed to evaluate the contribution of an instructional module to the identification of the number and characteristics of lymph nodes by undergraduate nursing students. We consider the hypothesis that the scores of correct answers in evaluating the characteristics and the identification of the lymph nodes will be higher after using the instructional module.

## Method

An experimental study, since it presents an educational intervention of undergraduate nursing students, with two groups, experimental and control, randomized and with masking^(^ 20 ^)^, carried out in a public higher education institution in the State of São Paulo. Considering that the study does not portray health-related intervention to modify a biomedical or health-related outcome of the patient and in view of the object, the objective and the study participants, no registration was made on a clinical registration platform. This practice is in line with the International Committee of Medical Journal Editors (ICMJE)^(^ 21 ^)^.

The population consisted of students enrolled in the Semiology and Semiotechnics discipline. All the students who expressed an interest were included, respecting the following inclusion criteria: having attended the expository-dialog theory class that addressed the lymphatic system, its function, organs and structures that compose it, location of ganglion chains, most common characteristics and changes; having participated in the practical activity of demonstrating inspection and palpation of the different chains and ganglia, as well as the position of the examiner’s hands to perform the palpation in the respective places; and having carried out the practical activity of these skills in a teaching laboratory, on healthy individuals. Such activities are those provided for in the discipline, on the evaluation of the lymphatic system; totaling nearly five hours and preceding the hospital practice. The exclusion criterion was failure to perform any of the research activities. It was adopted that the students who obtained 80% or more of total correct answers in the initial phase of the study would not have their data computed; however, none achieved such a level of correct answers.

As an intervention, the instructional module was used, consisting of a prototype for training lymph node palpation and of a guide instrument for recording the findings of palpation, constructed by the author. The module was validated with nursing experts and students, being considered useful for teaching this theme (75%) and capable of representing the characteristics of the lymph nodes (85%); both the prototype and the registration guide instrument were positively evaluated^(^ 22 ^)^.

The prototype used in the intervention was made up by a quadrangular box with five structures that simulated different lymph nodes in terms of size, consistency, mobility and coalescence. These characteristics were selected because they are the main ones in an evaluation of lymph nodes^(^ 16 ^)^. The empty spaces between the structures were filled in and the box covered with materials that provide a similar consistency to the subcutaneous and cutaneous tissues. The lateral edges of the box contained letters (A to K) and the upper and lower edges numbers (1 to 11) that allowed identifying the location of each structure. Lymph nodes I and II were hard and had nearly two centimeters in size, the first being mobile and the second fixed with a more elongated shape than the others; lymph node III was fixed, of hardened consistency, with nearly five centimeters, unlike the others, showed coalescence; lymph node IV had a soft consistency and was fixed; and lymph node V, the smallest of all, with nearly one centimeter, was fixed with a hardened consistency.

The guide instrument for recording the findings in the intervention, part of the instructional module, contained the guidelines for the development of the task (palpation) and for recording the results. The students were invited to perform the palpation considering the following instructions: size - palpate the lymph node and feel the limits of its structure and try to establish the size in centimeters; consistency - feel the degree of density that the lymph node has, if it is softened or hardened; mobility - try to slide the lymph node over the surface of the prototype and check if it is fixed or mobile; coalescence - palpate the lymph node and feel the delimitation of its shape, check if it is an isolated body or if it is adhered to other structures.

As for the record, they should describe the number of lymph nodes found in the prototype and their characteristics: location (report the reference coordinates of each lymph node), size (less than 0.5 cm, between 0.5 cm and 1.0 cm, between 1.0 cm and 2.0 cm, and greater than 2.0 cm), consistency (softened and hardened), mobility (mobile and non-mobile) and coalescence (coalescent and non-coalescent). Furthermore, the students could, at their discretion, graphically represent the location, size and shape of each lymph node identified in the prototype.

The outcomes considered in this study were the identification of the lymph nodes and the description of the characteristics of each one of them, in the two moments of data collection (initial and final phases). To record the outcomes, an instrument consisting of an identification sheet with the following data reported by the participant was used: name, date of collection, gender and date of birth. It also contained guidelines for the participant to carry out the activity, that is, it was highlighted that the student should identify the lymph nodes present in the prototype and freely describes the characteristics of each of them, in the blank space on the sheet. This instrument was used for the two collection moments, one copy for each stage.

For the study data collection protocol, another two prototypes were built, called Prototype A - initial evaluation and Prototype B - final evaluation, respectively, similar to the one described above, containing the same number and characteristics of the structures, but with a different location. The modification of the location of the lymph nodes in the three types of prototypes was intended to reduce possible bias.

All the students individually performed the initial assessment of the structures contained in Prototype A and performed free registration of the number and characteristics of the identified lymph nodes (initial collection). Only the students from the Experimental Group (EG) performed the intervention with the instructional module for the purposes of the study. Then, all the students re-evaluated the structures contained in Prototype B and performed the free registration of the number of characteristics of the identified lymph nodes (final collection). It should be noted that the Control Group (CG) was offered the instructional module after the final assessment, respecting the possibility of similar learning for all the participants. In addition, the answer template was made available by the author to all the interested participants, in a face-to-face meeting, after the end of the study data collection.

From the potential 109 students enrolled in the aforementioned discipline, 70 accepted the invitation to participate in the study; this was carried out by an auxiliary researcher in the classroom, at the end of the class following the one in which the content of the lymphatic system assessment was taught, with due prior authorization from the coordination of the discipline. After clarifying the purpose of the study and the scheduled stages, those students who were interested in participating were asked to record in a list their name, e-mail and preferred time for collection, among the hours offered. Confirmation was made via e-mail with collections scheduled for the week following the invitation.

Randomization was performed by a researcher, without previous contact with students, on an online platform found in the *www.random.org* ^®^ domain, where blocks of ten in ten numbers were selected; the operation was repeated until the number of potential participants was completed. The students were allocated to the groups according to their order of arrival and sent to the relevant laboratory, respecting the pre-established sequence.

Data collection was performed in the institution’s laboratories, during non-school hours; participation in the activity was expected to be carried out with a mean duration of up to 40 minutes. In the laboratory, sequences of stations were created, separated by a curtain, each containing a prototype and the appropriate instrument for each stage. The CG students conducted the initial evaluation with palpation of Prototype A and initial free registration (initial station), went to the next station and performed the same procedures of palpation and registration using Prototype B for the final evaluation (final station); afterwards, they got to know the instructional prototype (intervention station). The EG students conducted the initial evaluation with palpation of Prototype A and free registration (initial station), went to the intervention station and developed the activities of the instructional module and, subsequently, went to the final station and performed the palpation of Prototype B and final free registration. In each sequence, one student at a time was allowed to enter.

Masking occurred for the researcher who evaluated the outcomes and for those who performed the statistical analyses.

For data analysis, first, a template was created containing the correct information on the number, location and characteristics of the lymph nodes present in each of prototypes A and B. Afterwards, a researcher analyzed the students’ answers and inserted them in the data categorization instrument; this contained the notes of correct or incorrect answers for the variables of interest. An instrument was filled out for each participant and for each phase. This process, repeated three times, showed no data divergences.

The data obtained were inserted and analyzed in the R computer program, version 3.6.3^(^ 23 ^)^. For the gender and age variables, descriptive statistics were used; for the age variable, the mean and median were established and standard deviation was used as a measure for dispersion. Absolute and percentage frequencies were used for the categorical variables. Regarding the assessment of homogeneity (between groups) by gender and age, the Fisher’s exact and Mann-Whitney’s tests were used, respectively. The mixed or random effects model was used to analyze longitudinal data with a hierarchical structure, incorporating the dependence^(^ 24 ^)^.

The correctness (yes/no) of the variables identifying the presence, size, consistency, mobility, coalescence and location of each lymph node (I, II, III, IV and V) was evaluated, according to the study phase (initial and final) in each group (CG and EG). For these variables, it was assumed that they followed a binomial distribution.

In this model, the presence of a random effect was assumed, with normal distribution, only for the intercept of the model. In all the adjustments and tests performed, a significance level of 5% (α=0.05) and the use of the R Program^(^ 22 ^)^ were adopted.

The project was approved by the Institution’s Ethics Committee in Research with human beings, opinion No. 1,549,804. The Free and Informed Consent Term (FICT), in two copies, was obtained from all the participants.

## Results

**Figure 1:**
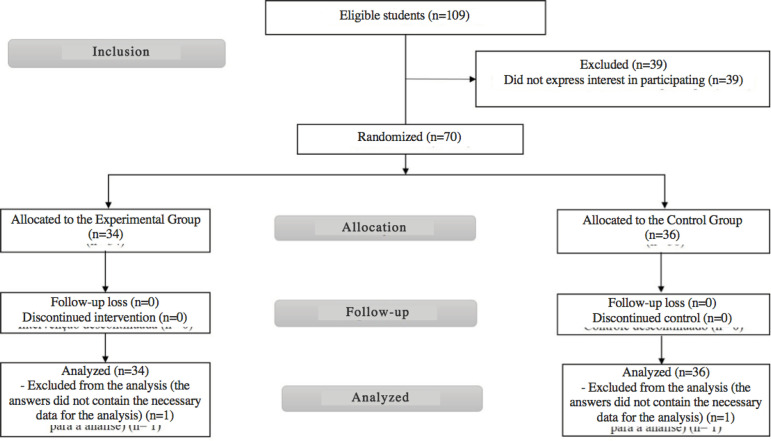
Flowchart of inclusion, allocation, follow-up and analysis of the participants, adapted from CONSORT^(^ 25 ^)^. Ribeirão Preto, SP, Brazil, 2016-2017

Among the 70 students who accepted the invitation, 34 were allocated to the EG and 36 to the CG; two study participants were excluded, one from each group, as they did not record the information requested to attain the activity proposal. The flowchart of inclusion, allocation, follow-up and analysis of the participants is shown below (Figure 1).

Of the 68 participants, 61 (89.71%) were female, 33 (48.53%) from the CG and 28 (41.18%) from the EG. The total mean age was 20.63 (standard deviation 1.58), being 20.71 (standard deviation 1.53; minimum of 19 and maximum of 25 years old) in the CG and 20.54 (standard deviation 1.63; minimum of 19 and maximum of 24 years old) in the EG; with a median of 20 in both. Homogeneity (between groups) was obtained for the gender (p=0.0920) and age (p=0.3260) variables.

In the initial phase of the study, when considering the students’ baseline knowledge, seven (10.29%), two from the CG (5.71%) and five from the EG (15.15%), referred all the characteristics of the lymph nodes found in the prototype used. It is noteworthy that almost half of the students (48.53%), 19 from the CG (54.29%) and 14 from the EG (42.42%), mentioned the existence of one extra lymph node than the number actually contained in the prototype at this stage.

When considering the percentages of correct answers regarding the identification of the number of lymph nodes and the characteristics of each one when palpating the prototypes, it is noticed that, in the initial phase, both groups obtained a similar response pattern for most of the lymph nodes (Table 1).

**Table 1:** Identification of the number and characteristics of the lymph nodes, by group, in the different phases of the study (N=68). Ribeirão Preto, SP, Brazil, 2016-2017

Variable	*CG		^†^EG		Total		*CG		^†^EG		Total		
	Initial		Initial		Initial		Final		Final		Final		
	n	%	n	%	n	%	n	%	n	%	n		%
*Number of lymph nodes:*													
	7	20.00	5	15.15	12	17.65	13	37.14	16	48.48		29	42.65
*Characteristics by lymph node*													
*Size*													
Lymph node I	17	48.57	14	42.42	31	45.59	16	45.71	24	72.73		40	58.82
Lymph node II	10	28.57	10	30.30	20	29.41	13	37.14	22	66.67		35	51.47
Lymph node III	2	5.71	4	12.12	6	8.82	3	8.57	18	54.55		21	30.88
Lymph node IV	10	28.57	9	27.27	19	27.94	15	42.86	25	75.76		40	58.82
Lymph node IV	3	8.57	4	12.12	7	10.29	7	20.00	13	39.39		20	29.41
*Consistency*													
Lymph node I	16	45.71	20	60.61	36	52.94	15	42.86	28	84.85		43	63.24
Lymph node II	11	31.43	10	30.30	21	30.88	13	37.14	23	69.70		36	52.94
Lymph node III	15	42.86	19	57.58	34	50.00	22	62.86	32	96.97		54	79.41
Lymph node IV	13	37.14	13	39.39	26	38.24	12	34.29	27	81.82		39	57.35
Lymph node IV	2	5.71	3	9.09	5	7.35	3	8.57	15	45.45		18	26.47
*Mobility*													
Lymph node I	3	8.57	6	18.18	9	13.24	14	40.00	25	75.76		39	57.35
Lymph node II	9	25.71	6	18.18	15	22.06	10	28.57	27	81.82		37	54.41
Lymph node III	9	25.71	11	33.33	20	29.41	9	25.71	33	100.00		42	61.76
Lymph node IV	6	17.14	3	9.09	9	13.24	9	25.71	23	69.70		32	47.06
Lymph node IV	1	2.86	2	6.06	3	4.41	3	8.57	16	48.48		19	27.94
*Location*													
Lymph node I	29	82.86	29	87.88	58	85.29	29	82.86	33	100.00		62	91.18
Lymph node II	22	62.86	25	75.76	47	69.12	28	80.00	30	90.91		58	85.29
Lymph node III	28	80.00	29	87.88	57	83.82	30	85.71	33	100.00		63	92.65
Lymph node IV	25	71.43	26	78.79	51	75.00	28	80.00	31	93.94		59	86.76
Lymph node IV	4	11.43	7	21.21	11	16.18	11	31.43	17	51.52		28	41.18
*Coalescence*													
Lymph node III	8	22.86	9	27.27	17	25.00	5	14.29	27	81.82		32	47.06

* CG = Control group (n=35);

† EG = Experimental group (n=33)

As for the identification of the number of existing lymph nodes, there was an increase in correct answers for both groups, with greater frequency for the EG (Table 1).

When specifically examining the five characteristics of the lymph nodes, the highest percentages of correct answers in the study were for the location variable, for all lymph nodes; the consistency and size variables followed, with a percentage of varied correct answers and coalescence, present only in lymph node III. The characteristic with the lowest percentage of correct answers was mobility. Lymph node V showed lower rates of correct answers for most of the characteristics analyzed (Table 1).

In general, in the initial phase, the groups obtained a similar pattern in the responses when considering the percentage of correct answers of the five characteristics for most of the evaluated lymph nodes. In the final phase, there was an increase in the correct answers in the groups, for most of the variables related to the lymph nodes, but in different percentages. The reduction in the number of correct answers by a CG student in the final stage stands out for the size (lymph node I) and consistency (lymph nodes I and IV) variables, and for another three for the coalescence variable (lymph node IV) (Table 1).

**Figure 2:**
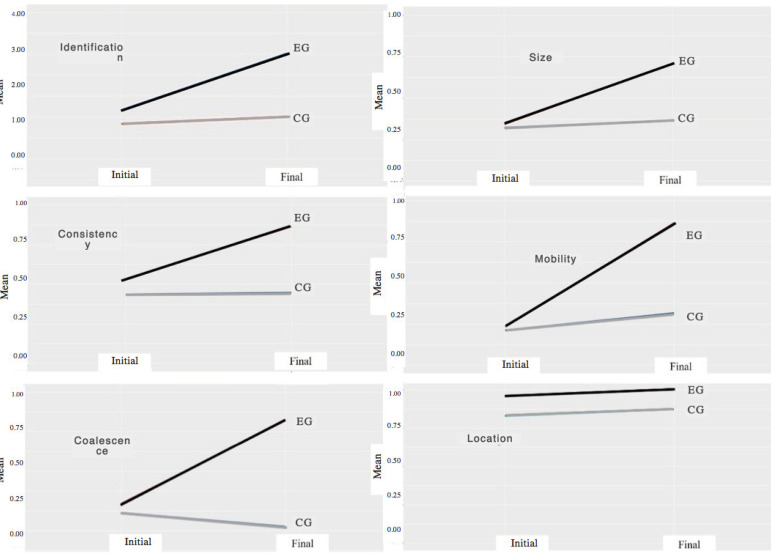
Interaction between time (initial and final) and groups (CG and EG) for the identification and characteristics variables (size, consistency, mobility, coalescence and location) of the lymph nodes. Ribeirão Preto, SP, 2016-2017

The graphical representation of the interactions of the identification of the characteristics of the lymph nodes evaluated in the groups (CG and EG) and in the times (initial and final) allowed perceiving the similarity between the correct answers at the beginning of the study and their difference in the final phase, especially for the EG, for most of the characteristics studied (Figure 2).

The analysis of the effect of the interactions of the variables of interest on the groups and the times of the study is expressed as follows (Table 2).

**Table 2:** Linear regression analysis of mixed effects for the characteristics of the lymph node according to the groups (N=68). Ribeirão Preto, SP, Brazil, 2016-2017

Variables	Estimate	Standard error	z-value	p-value
*Identification*				
Intercept	5.708	1.015	5.627	0.000
Experimental Group	0.277	0.658	0.421	0.674
Final phase	1.832	0.368	4.986	0.000
Lymph node II	-3.743	0.915	-4.093	0.000
Lymph node III	-1.241	0.970	-1.279	0.201
Lymph node IV	-3.242	0.912	-3.556	0.000
Lymph node IV	-8.205	1.080	-7.599	0.000
*Size*				
Intercept	-0.985	0.619	-1.592	0.112
Experimental Group	0.346	0.806	0.429	0.668
Final phase	0.340	0.374	0.907	0.364
Lymph node II	-0.539	0.393	-1.372	0.170
Lymph node III	-3.062	0.450	-6.802	0.000
Lymph node IV	-0.432	0.387	-1.115	0.265
Lymph node IV	1.008	0.702	1.435	0.151
Experimental Group: Final phase	2.899	0.614	4.719	0.000
*Consistency*				
Intercept	-0.578	0.548	-1.054	0.292
Experimental Group	0.980	0.725	1.352	0.176
Final phase	0.130	0.349	0.373	0.709
Lymph node II	-0.733	0.377	-1.942	0.052
Lymph node III	0.773	0.377	2.049	0.041
Lymph node IV	-0.435	0.371	-1.172	0.241
Lymph node IV	-0.499	0.568	-0.878	0.380
Experimental Group: Final Phase	2.832	0.562	5.037	0.000
*Mobility*				
Intercept	-2.784	0.565	-4.926	0.000
Experimental Group	0.663	0.657	1.009	0.313
Final phase	0.961	0.387	2.480	0.013
Lymph node II	0.750	0.399	1.879	0.060
Lymph node III	1.051	0.386	2.721	0.007
Lymph node IV	-0.290	0.398	-0.730	0.466
Lymph node IV	0.190	0.566	0.336	0.737
Experimental Group: Final phase	3.361	0.591	5.684	0.000
*Coalescence*				
Intercept	-2.427	1.061	-2.288	0.022
Experimental Group	0.626	1.161	0.539	0.590
Final phase	-1.254	0.988	-1.269	0.205
Experimental Group: Final phase	5.950	1.935	3.075	0.002
*Location*				
Intercept	10.146	2.382	4.260	0.000
Experimental Group	1.492	1.753	0.851	0.395
Final phase	5.126	1.782	2.877	0.004
Lymph node II	1.074	1.439	0.747	0.455
Lymph node III	-0.255	1.141	-0.223	0.823
Lymph node IV	-0.024	1.295	-0.019	0.985
Lymph node IV	-0.495	2.288	-0.217	0.829

Regarding the identification of lymph nodes, Figure 2 suggests a difference in correct answers between the two phases of the study, with an apparent predominance for the EG; regression analysis shows that there was a difference, observed by the improvement in the final evaluation for lymph nodes II, with nearly 2 cm, hardened, fixed consistency and with an elongated shape (p<0.0001); IV, with softened and fixed consistency (p=0.0004); and V, nearly 1 cm, hardened and fixed consistency (p<0.0001) for the students in general.

In relation to the size and consistency variables, the effect of group interaction in the final stage stands out, observed by the difference in correct answers between the groups for this phase, with better performance by the EG (Figure 2); this fact is evidenced in the regression analysis (p≤0,0001); also, the rate of correct answers on the size of lymph node III showed a difference (p≤0,0001), with an improvement in its description, and the consistency of the same lymph node also showed a significant increase in correct answers (p=0.0405) (Table 2).

As for mobility, the interaction (Figure 2) between the EG and CG groups in the two periods studied illustrates the similarity between the correct answers in the initial phase, in the same way that it highlights the difference between them in the final phase, with the EG obtaining higher success rate. The regression analysis (Table 2) shows that there was a difference in the number of correct answers of lymph node mobility, comparing the initial and final phases (p=0.0131) with a difference between the groups (p<0.0001), that is, a higher rate of correct answers in the EG. The answers obtained for lymph node III (p=0.0065) contributed to this difference.

As for coalescence, the interaction between the groups (EG and CG), in the initial and final times (Figure 2), shows that, although there was some similarity between the correct answers at the beginning of the study, this setting changed, with the EG having higher means, a result also confirmed by the regression analysis (p=0.0021) (Table 2).

As for the location (Figure 2), it is noted that, already in the initial phase, the EG obtained higher mean scores than the CG, following this trend for the final phase. The variables evaluated in the regression analysis (Table 2) confirm that there was a difference, for both groups, over the study phases (p≤0.0001).

In addition, the analyses performed it possible to verify, by group and study times, the Odds Ratios (ORs) to carry out the evaluation of the studied variables, except coalescence. The interaction effects for the lymph node identification and location variables were not significant over time (Table 3).

**Table 3:** Odds Ratio (OR) parameters by time and group for identification and lymph node characteristics. Ribeirão Preto, SP, Brazil, 2016-2017

Variable		Contrast	OR*	SE^†^	z.ratio	LL^‡^	UL^§^	p-value
	*Time*							
Identification	Initial	EG^||^/CG^¶^	1.32	0.87	0.42	0.30	5.76	1.0000
	Final	EG^||^/CG^¶^	1.32	0.87	0.42	0.30	5.76	1.0000
Consistency	Initial	EG^||^/CG^¶^	2.66	1.93	1.35	0.52	13.53	0.3527
	Final	EG^||^/CG^¶^	45.26	35.65	4.84	7.74	264.54	0.0000
Location	Initial	EG^||^/CG^¶^	4.45	7.79	0.85	0.09	140.74	0.7894
	Final	EG^||^/CG^¶^	4.45	7.79	0.85	0.09	140.74	0.7894
Mobility	Initial	EG^||^/CG^¶^	1.94	1.28	1.01	0.45	8.47	0.6259
	Final	EG^||^/CG^¶^	55.95	37.51	6.00	12.45	251.60	0.0000
Size	Initial	EG^||^/CG^¶^	1.41	1.14	0.43	0.24	8.58	1.0000
	Final	EG^||^/CG^¶^	25.64	21.35	3.90	3.92	160.62	0.0002
	*Group*							
Identification	CG^¶^	Final/Initial	6.25	2.30	4.99	2.74	14.24	0.0000
	EG^||^	Final/Initial	6.25	2.30	4.99	2.74	14.24	0.0000
Consistency	CG^¶^	Final/Initial	1.14	0.40	0.37	0.52	2.49	1.0000
	EG^||^	Final/Initial	19.34	8.63	6.64	7.11	52.59	0.0000
Location	CG^¶^	Final/Initial	168.27	299.80	2.88	3.12	13970.20	0.0080
	EG^||^	Final/Initial	168.27	299.80	2.88	3.12	13970.20	0.0080
Mobility	CG^¶^	Final/Initial	2.61	1.01	2.48	1.10	6.23	0.0263
	EG^||^	Final/Initial	75.34	35.67	9.13	26.07	217.68	0.0000
Size	CG^¶^	Final/Initial	1.40	0.53	0.91	0.62	3.29	0.7285
	EG^||^	Final/Initial	25.49	12.65	6.52	8.30	76.25	0.0000

* OR = Odds ratio;

† SE = Standard error;

‡ LL = Confidence interval lower limit;

§ UL = Confidence interval upper limit;

|| EG = Experimental group;

¶ CG = Control group

When comparing the times, in the final phase, the EG was 45.26 times more likely to carry out the consistency assessment, 55.95 times more likely to carry out the mobility assessment, and 25.64 times more likely to carry out the consistency assessment, than the CG (Table 3).

When comparing the two study groups, in the EG, the participants in the final phase were 19.3 times more likely to assess consistency, 75.33 times more likely to assess mobility, and 25.49 times more likely to assess size than in the initial phase. In the CG, the participants in the final phase were 2.61 times more likely to assess mobility than in the initial phase (Table 3).

Furthermore, at the end of the study, the participants in general were 6.25 times more likely to identify the lymph nodes and 168.27 times more likely to assess the location than in the initial phase (Table 3).

Although all the students were more likely to identify the lymph nodes and their location, it is noteworthy that the use of the instructional module resulted in greater chances of assessing three of the lymph node characteristics (mobility, size and consistency) more completely and correctly by the EG in relation to the CG (p<0.05) in the final phase.

## Discussion

This study evaluated the contribution of an instructional module to the identification and description of lymph node characteristics by nursing students.

Knowledge about the development and registration of the physical examination, object of this study, is the focus of interest in different areas, given its relevance. In a study on the evaluation of the physical examination record, performed with medical students and interns, the record was considered incomplete; the presence of the description on the evaluation for the lymph nodes was performed only in 16.6%. The authors reiterate that 35% of the patients examined had infectious and/or autoimmune disease and 15%, malignant neoplasm, which reinforces the importance of such an assessment and of a quality record^(^ 26 ^)^. Peripheral lymphadenopathy is common evidence in benign situations, but it can be related to malignant events. Therefore, information on location, size, consistency, mobility and presence of pain contribute to clinical reasoning^(^ 27 ^)^.

In nursing, the quality of the records was also assessed; according to the researchers, only 22.8% of the records examined were considered complete^(^ 28 ^)^. A similar study carried out in an Intensive Care Unit verified a high percentage of non-registration of the physical examination^(^ 29 ^)^. Such data reinforce the importance of implementing teaching strategies for the adequate recording of patient data.

The literature points out that, in relation to the student’s good performance in the long run, teaching in laboratory skills is suitable for teaching easier skills; in this sense, the proposal with the use of the instructional module (prototype and guide instrument for evaluation) proved to be promising. Using a simplified guide for teaching physical examination was also useful when comparing the performance of medical students. The authors observed improvements in several items in relation to the complete evaluation, after training with the guide, including the performance of the lymph node palpation procedure, which showed a significant improvement (p<0.01)^(^ 30 ^)^.

In this study, the results of the initial phase were similar for both groups for most of the variables analyzed; the students in general showed better results in their answers in the final phase of the study. This is justified by the theory adopted^(^ 15 ^)^, which considers that contact with a situation of experience, after exposure to theoretical content, alone generates learning (Figure 2). Such results are reinforced by those obtained by the students in general at the end of the study, who were more likely to identify the lymph nodes and to assess the location than in the initial phase (Table 3).

The theory of experiential learning^(^ 15 ^)^ recommends that, for learning to be more effective, the students must have contact with the educational resource several times; in this sense, in our study, although the EG students have showed satisfactory results with the handling of the instructional module with only one exposure, multiple exposures could have better success rates. From this point of view, if it is considered that handling the evaluation module itself could have interfered with learning, attention is drawn to the reduction of the rates of correct answers for the location and consistency variables by a student in the CG, subjected to reduced attention or motivation. In the case of a reduction in the number of correct answers for the coalescence variable, in the final phase, in three students in the CG, we considered the possibility that the characteristic of the lymph node might have led to error, since there was an increase in the improper identification of the existence of a lymph node (Table 1).

The literature points out that quality (characteristics such as heat, underlying erythema, sensitivity, mobility, consistency and fluctuation) and lymph node size are essential for clinical evaluation^(^ 16 ^-^ 19 ^)^. In this sense, even though the specificities of the present prototype favored the learning of part of these characteristics, it can be said that it was promising, given that the results of the final phase of the study showed an increase in the number of correct answers for the characteristics of size, consistency, mobility and coalescence (p<0.05), when compared to the initial phase, by the EG participants. The relevance of the purpose of teaching ganglion palpation is also added; this is based on the premise that, among the various methods currently available, physical palpation, ultrasound and computed tomography are minimally invasive, easily available, and can be performed routinely^(^ 31 ^)^.

When looking at the inclusion criterion, which required prior participation by the students in theoretical teaching in the classroom of the content covered in this study, a contribution to the discipline is envisaged. In general, the students do not have the experience of handling altered lymph node structures; this may have made it difficult for the students to complete all the learning stages^(^ 15 ^)^ in the prior formal education, as depicted in the initial study results. Therefore, the palpation experience, together with the recording of the characteristics, such as that performed in the proposed intervention, aims to increase the sedimentation of knowledge before the student has experienced the clinical practice; in this sense, the module will contribute to complementing the teaching-learning process. As already described, the theory-practice intersection, when experienced in the academy, corroborates with the construction of competences and skills necessary for the development of a better quality care practice^(^ 5 ^)^.

Finally, even though the strategy used is a low-fidelity simulator, it is considered that its use as an alternative teaching resource stimulates and contributes to learning, as pointed out both by the experience of authors in the medical field when using simple anatomical models in teaching human anatomy^(^ 32 ^)^ and by that of those in the nursing area who support the use of low-fidelity simulators associated with simulated patients, with dramatization support^(33)^. The high Odds Ratios for the EG student to correctly assess the characteristics of consistency, mobility and size in relation to the CG obtained in the present study reinforce the contribution of the prototype employed.

Possible limitations in the planning and development of the study may have occurred, in relation to the craft characteristics of the instructional material, to the masking of only the evaluator and the researcher who carried out the statistical treatment, as well as to the number of participants, comprising 64% of the potential students. Another aspect to be considered concerns the non-inclusion of a structured evaluation on the learning experience, despite the highly positive considerations, as described when the instructional module was validated.

## Conclusion

Using an instructional module contributed to increase the knowledge of nursing students on the assessment of lymph node size, consistency, mobility and coalescence.

The high Odds Ratios for the student exposed to the intervention to make a correct assessment of the lymph node characteristics obtained in this study reinforce the contribution of using the instructional module, still in a clinical practice laboratory, for the sedimentation of learning, essential for the student’s diagnostic and therapeutic clinical reasoning.
